# Who Is Who in Adenosine Transport

**DOI:** 10.3389/fphar.2018.00627

**Published:** 2018-06-14

**Authors:** Marçal Pastor-Anglada, Sandra Pérez-Torras

**Affiliations:** ^1^Molecular Pharmacology and Experimental Therapeutics, Department of Biochemistry and Molecular Biomedicine, Institute of Biomedicine, University of Barcelona, Barcelona, Spain; ^2^Oncology Program, National Biomedical Research Institute on Liver and Gastrointestinal Diseases – CIBER ehd, Institut de Recerca Sant Joan de Déu, Barcelona, Spain

**Keywords:** adenosine, transporters, CNT, ENT, purinergic signaling

## Abstract

Extracellular adenosine concentrations are regulated by a panel of membrane transporters which, in most cases, mediate its uptake into cells. Adenosine transporters belong to two gene families encoding Equilibrative and Concentrative Nucleoside Transporter proteins (ENTs and CNTs, respectively). The lack of appropriate pharmacological tools targeting every transporter subtype has introduced some bias on the current knowledge of the role of these transporters in modulating adenosine levels. In this regard, ENT1, for which pharmacology is relatively well-developed, has often been identified as a major player in purinergic signaling. Nevertheless, other transporters such as CNT2 and CNT3 can also contribute to purinergic modulation based on their high affinity for adenosine and concentrative capacity. Moreover, both transporter proteins have also been shown to be under purinergic regulation via P1 receptors in different cell types, which further supports its relevance in purinergic signaling. Thus, several transporter proteins regulate extracellular adenosine levels. Moreover, CNT and ENT proteins are differentially expressed in tissues but also in particular cell types. Accordingly, transporter-mediated fine tuning of adenosine levels is cell and tissue specific. Future developments focusing on CNT pharmacology are needed to unveil transporter subtype-specific events.

## Introduction

Oscillation of extracellular adenosine levels is physiologically relevant because this nucleoside is the agonist of four P1 receptors known to modulate many biological functions (Fredholm et al., 2011; Burnstock, 2017). Indeed, adenosine concentrations in the extracellular milieu are determined by the balance between its appearance and its removal. In most cases adenosine appearance is the result of the sequential metabolic action of various ecto-nucleotidases on nucleotide precursors, ATP being the first nucleotide in this cascade (Dos Santos-Rodrigues et al., 2014; Nguyen et al., 2015; Pastor-Anglada et al., 2018). However, in particular cell types, there is experimental evidence supporting the possibility of adenosine also being released from cells (Almeida et al., 2003). Extracellular adenosine disposal is similarly mediated by either metabolism, Adenosine Deaminase (ADA) being the enzyme responsible for its conversion into inosine, or by its uptake into cells, where it is likely to be metabolized and trapped as AMP after being phosphorylated by Adenosine Kinase (ADK). The relative contribution of each particular mechanism to the oscillations of adenosine levels may be cell-specific and dependent upon the tissue microenvironment, but is not well-known, although some attempts to address this issue have been done (Nguyen et al., 2015). In any case, adenosine removal from the extracellular milieu is likely to play a major role in regulating adenosine concentrations.

Adenosine cannot freely permeate biological membranes and its transport across them occurs via selected adenosine transporter proteins. Accordingly, transport processes are key modulators of extracellular adenosine disposal. In this contribution, we plan to provide an updated and critical view on the particular transporter subtypes likely to mediate adenosine transport. Evidence supporting the link between transport processes and purinergic signaling will be also discussed.

## Adenosine Transport Mechanisms

All adenosine transporters identified so far belong to the SoLute Carrier (*SLC*) superfamily, in particular, to gene families *SLC28* and *SLC29* (Young et al., 2013; Young, 2016; Pastor-Anglada et al., 2018). *SLC28* genes encode three transporter subtypes known as human Concentrative Nucleoside Transporters 1, 2, and 3 (hCNT1, hCNT2, and hCNT3). The *SLC29* family has four members, thereby resulting in four transporter subtypes, known as human Equilibrative Nucleoside Transporters 1, 2, 3, and 4 (hENT1, hENT2, hENT3, and hENT4). Evidence for additional transporter subtypes, generated by mRNA splicing has been provided for hCNT3 and hENT2, in both cases leading to shorter proteins than their corresponding wild type transporters. Nevertheless, in all cases these small variants appear to be localized in intracellular compartments (Errasti-Murugarren et al., 2009; Grañé-Boladeras et al., 2016) and are unlikely to play any significant role in purinergic signaling. Nevertheless, it has been shown that hENT2 splice variants can regulate wild type hENT2 abundance and function at the plasma membrane (Grañé-Boladeras et al., 2016).

The type of translocation processes implicated in adenosine transport (i.e., “concentrative” versus “equilibrative”) and the affinity binding of adenosine to its transporter proteins are key determinants of adenosine transport efficacy.

hCNTs are obligatory inward transporters which take advantage of the sodium gradient to accumulate nucleosides in the cells. Nucleosides and sodium are co-transported with translocation stoichiometry 1:1 (hCNT1 and hCNT2) and 1:2 (hCNT3). Indeed, those CNT proteins showing the ability to transport adenosine are excellent candidates to promote adenosine disposal from the extracellular milieu due to their concentrative capacity. hENTs are potentially bidirectional, vectorial transport being determined by the nucleoside concentration gradient across the membrane. Nevertheless, it is probable that in some circumstances, functional coupling of adenosine influx with its intracellular phosphorylation by ADK enables cells to trap this nucleoside as AMP thereby building up a transmembrane adenosine gradient which will favor unidirectional import of adenosine. It is not known whether adenosine release via these transporters can be explained by some sort of inefficient, not necessarily uncontrolled coupling between metabolism and transport.

As introduced above, affinity is also a critical parameter when discussing the adenosine transport capacity of each nucleoside transporter subtype. Reported physiological adenosine concentrations are very low, often below 1 μM (Fenton and Dobson, 1992; Espinoza et al., 2011; Rose et al., 2011; Westermeier et al., 2011), although under certain conditions, such as hypoxia or in tumor microenvironments where ATP levels can increase considerably, adenosine can also accumulate above normal physiological concentrations (Blay et al., 1997) reviewed in de Andrade Mello et al. (2017) and Di Virgilio and Adinolfi (2017). As shown in **Table 1**, apparent Km values for adenosine vary among transporter subtypes, although some intrinsic variability is observed for the same transporter subtype, probably as a result of the experimental set used to calculate this parameter.

**Table 1:** Affinity constants of human adenosine transporters.

Gene name protein	Experimental model	Type of assay	Af. ct. (μM)	Reference
*SLC29A1* ENT1	Human Erythroleukemia K562 cells. Endogenous transporters. Human Umbilical Vein Endothelial Cells (HUVEC). Endogenous transporters. Placental microvascular endothelial cells. Endogenous transporters. Heterologous expression in PK15NTD (Nucleoside Transporter Deficient) cells.	Adenosine influx Adenosine influx Adenosine influx Adenosine influx	75 32 53 59 61 82 40	Boleti et al., 1997 Celis et al., 2017 Casanello et al., 2005 Muñoz et al., 2006 Salomón et al., 2012 Escudero et al., 2008 Ward et al., 2000
*SLC29A2* ENT2	Human Umbilical Vein Endothelial Cells (HUVEC). Endogenous transporters. Placental microvascular endothelial cells. Endogenous transporters. Heterologous expression in PK15NTD (Nucleoside Transporter Deficient) cells.	Adenosine influx Adenosine influx Adenosine influx	49 102 77 98 140	Celis et al., 2017 Muñoz et al., 2006 Salomón et al., 2012 Escudero et al., 2008 Ward et al., 2000
*SLC29A3* ENT3	hENT3AA, mutated to reach the plasma membrane in *Xenopus laevis* oocytes. Δ36hENT3, deleted to reach the plasma membrane in *Xenopus laevis* oocytes.	Adenosine influx pH 5.5 Adenosine influx pH 5.5	1860 1620 1800	Baldwin et al., 2005 Rahman et al., 2017 Kang et al., 2010
*SLC29A4* ENT4	Expression in *Xenopus laevis* oocytes.	Adenosine influx pH 5.5	780	Barnes et al., 2006
*SLC28A2* CNT2	Expression in *Xenopus laevis* oocytes.	Adenosine influx Adenosine influx Electrophysiology Electrophysiology	8 6 18 23	Yao et al., 1996 Che et al., 1995 Larráyoz et al., 2006 Li et al., 2001
*SLC28A3* CNT3	Expression in *Xenopus laevis* oocytes Heterologous expression in yeast Heterologous expression in HeLa cells	Adenosine influx Electrophysiology Adenosine influx Adenosine influx	15 18 2.2 2.4	Ritzel et al., 2001 Gorraitz et al., 2010 Damaraju et al., 2005 Errasti-Murugarren et al., 2008

*Interaction of nucleoside transporters with adenosine has been addressed either by determining the influx of radiolabeled adenosine into cells or, for hCNTs, by monitoring adenosine-induced Na^+^currents in Xenopus laevis oocytes expressing a particular CNT subtype protein. Apparent affinity constants (Af. ct.) are all given in μM for a better comparison among all NT subtypes. Endogenous transporters refer to kinetic determinations performed using cell lines which express hENT1 and hENT2 endogenously, being the contribution of each subtype calculated by selectively inhibiting either hENT1 alone or both hENTs, as explained in the text. Adenosine uptake by hENT3 and hENT4 can only be measured at acidic pH. Kinetic determinations using the intracellular transporter hENT3 can only be performed if the wild type protein is modified in a way that sorting signals are blocked, thereby allowing the protein to reach the plasma membrane and determine function. Whether these relatively small structural alterations can significantly impact on adenosine affinity is not known. hCNT1 is not included in the table because, as explained in the text, even though some adenosine affinity constants have been reported, its translocation efficacy is extremely poor and we think it cannot be considered an adenosine transporter in a physiological context.*

Adenosine transport can be determined either by influx measurements of its radiolabeled form or, at least for CNT-type transporters, by electrophysiological means. In order to get accurate determinations of kinetic parameters it is important to overexpress a particular subtype on a nucleoside-transport null background, which indeed is very rare, although two mammalian non-commercial cell lines lacking CNT- and ENT-related activity had been engineered for this purpose (Mackey et al., 1998; Ward et al., 2000). On the other hand, determination of adenosine uptake kinetic constants of endogenous transporters is a big challenge. In general trends, most cell lines do not retain hCNT-related activity, because hCNT expression is highly dependent upon cell differentiation. Moreover, kinetic determinations in primary cells are not easy either because they co-express several transporter subtypes showing overlapping substrate selectivity.

Nucleoside uptake by each particular nucleoside transporter subtype cannot be determined directly. This is a limitation likely to result in experimental variability. CNT-mediated transport is Na-dependent, but uptake determinations in saturating sodium concentrations (normally 120 mM NaCl) incorporate CNT- and ENT-related transport as well a variable (often small) residual component likely to be associated with non-specific binding, to which even the support where the cells grow on can contribute to. Thus, uptake measurementsin the absence of sodium are required, being this cation often replaced by choline (120 mM Choline Cl). CNT-type activity can be calculated by subtracting uptake rates measured in the absence of Na from the total uptake activity measured in a Na-containing medium. Needless to say, uptake rates should be measured in initial velocity conditions for proper calculation of kinetic constants. This can be experimentally challenging, particularly when transporters are overexpressed. Under these conditions substrate uptake can be very fast and short uptake time points might be needed (seconds). As mentioned above, it is not correct to assume that endogenous ENT-type proteins are responsible for all apparent uptake activity measured in the absence of sodium. This means in practice that direct measurements of ENT-related activity cannot be performed either. Thus, ENTs must be pharmacologically inhibited to figure out their contribution to the remaining transport activity measured in sodium-free medium. Indeed, both plasma membrane ENTs (ENT1 and ENT2) can be simultaneously blocked by μM concentrations of dipyridamole and dilazep, whereas ENT1 can be selectively inhibited by nM concentrations of the nucleoside analog NBMPR (Young et al., 2013; Young, 2016; Pastor-Anglada et al., 2018). Accordingly, ENT1 activity corresponds to the NBMPR-sensitive component whereas ENT2 contribution to nucleoside uptake can be calculated by subtracting the ENT1 activity from the dipyridamole-sensitive component (ENT1 and ENT2 working simultaneously). At this moment, the reader can easily understand to what extent accurate measurements of endogenous nucleoside transport activity can be challenging in primary cells co-expressing all types of transporter proteins.

As introduced above, electrophysiology might be suitable for accurate kinetic measurements taking advantage of the fact that hCNT proteins are electrogenic when they translocate nucleosides and sodium. The two-electrode voltage clamp technique has been broadly used in transporter biology for this purpose (Lostao et al., 2000; Larráyoz et al., 2004; Smith et al., 2004; Slugoski et al., 2008; Gorraitz et al., 2010). The cRNA coding for a specific transporters is injected in *Xenopus laevis* oocytes and, in normal conditions, transporter function can be assessed after 2 days. Oocytes are clamped and inward sodium currents triggered by the addition of a particular hCNT substrate are recorded. Indeed, the intensity of the applied current to compensate for the transient depolarization associated with sodium influx, reflects transport activity. In this particular set up, initial velocity conditions can be easily achieved, endogenous activity is not interfering and currents may be a more direct way of measuring hCNT transport function than when using radiolabeled adenosine influx determinations. However, the oocyte membrane might show physicochemical properties different from mammalian plasma membranes. To what extent the membrane environment of a particular nucleoside transporter determines function and, eventually, substrate specificity is not really well-known. In this regard, when studying a novel polymorphic hCNT3 variant identified in our laboratory several years ago (Errasti-Murugarren et al., 2008), we observed that hCNT3 can indeed be found in different membrane microdomains, hCNT3 proteins located in lipid rafts being more active than the ones off rafts (Errasti-Murugarren et al., 2010).

There are many experimental issues which are likely to affect hCNT-related activity measurements and this may explain published variability in substrate specificity (adenosine affinity constants). Despite all these experimental issues, in general terms, it seems that the transporter proteins showing the highest affinity for adenosine are hCNTs, in particular, hCNT2 and hCNT3. In our hands, apparent Km values for adenosine in HEK293 cells expressing hCNT3 are the lowest reported so far among all nucleoside transporter proteins, 2.4 μM. Nevertheless, what makes hCNT3 an excellent candidate to modulate extracellular adenosine levels is the fact that it shows a huge capacity to concentrate nucleosides inside cells, because of its unique stoichiometry. Nevertheless, as will be discussed below, hCNT3 is not ubiquitously expressed, meaning that in some particular cell types hCNT2 would be the one to do the job.

The role of hCNT1 in adenosine regulation requires a more detailed explanation because there has been an argument in the past about whether or not this protein is an adenosine transporter. Apparent Km values for adenosine have been calculated for rCNT1 when it was expressed in oocytes and transport assays were performed using radiolabeled nucleosides (Yao et al., 1996). In this set up, an apparent Km of 26 μM was reported and discussed to be similar to the one calculated for uridine (37 μM) (Huang et al., 1994). Nevertheless, under these conditions the Vmax for uridine was 300 fold higher than the one calculated for adenosine (Yao et al., 1996). The same laboratory reported very low, almost negligible, substrate-induced Na-inward currents when using saturating concentrations of adenosine (100 μM) in oocytes expressing the human CNT1 ortholog (Smith et al., 2004). In our hands currents were undetectable using the same experimental approach (Larráyoz et al., 2004). In summary, we believe hCNT1 cannot be considered an adenosine transporter protein. However, we generated some evidence suggesting the possibility that adenosine can instead bind to the transporter protein without being translocated. Evidence for adenosine binding is quite consistent. Most sodium-coupled transporters, among them hCNTs, show some sodium leakage in the absence of the co-substrate (a nucleoside in our case). Leakage can be similarly measured as a current and is dependent upon membrane potential. Adenosine was shown to block what are called pre-steady state and steady state currents of the transporter protein associated with sodium-leakage (Larráyoz et al., 2004). This can be understood as the consequence of adenosine binding to the transporter protein. The physiological relevance of this event is not known.

Regarding ENT proteins, most available literature points to ENT1 and ENT2 as major players in the regulation of adenosine levels. Although apparent Km values are definitely higher (even much higher for hENT2) than the ones reported for hCNT2 and hCNT3, efficient coupling with adenosine phosphorylation would contribute to generate a huge transmembrane adenosine gradient which thermodynamically would favor influx via these transporter proteins. As discussed below, ENT1 is by far the most studied member within the *SLC29* gene family and different laboratories have provided consistent evidence supporting a role for this particular subtype in adenosine signaling. ENT3 is mostly localized in intracellular compartments (probably mitochondria and lysosomes) but, in any case, its affinity for adenosine seems to be low enough as to preclude any role for this transporter protein in adenosine regulation (Baldwin et al., 2005; Kang et al., 2010; Hsu et al., 2012; Rahman et al., 2017).

Similarly to CNT1, ENT4 also requires some detailed explanations, because its role in adenosine signaling is still on debate. ENT4 is evolutionarily distant from the other three members of the family (Young et al., 2013) and, when cloned and functionally expressed it was reported to show poor affinity for nucleosides, whereas it could translocate monoamine neurotransmitters such as dopamine and serotonin (Engel et al., 2004). In fact the laboratory that generated all this information claimed ENT4 to be renamed as PMAT, from Plasma Membrane Amine Transporter. Interestingly, ENT4/PMAT shows functional similarity with organic cation transporters (OCTs), which means that this protein can act as a polyspecific OCT as the *SLC22* gene members encoding for hOCT1, 2, and 3. Some common substrate structural determinants between nucleoside transporters and OCTs can be hypothesized. Indeed the three hOCT proteins can efficiently translocate the antiviral nucleoside analog lamivudine. Moreover, they can also interact with several other nucleoside-based antiviral drugs such as zidovudine, abacavir, and others (Minuesa et al., 2009). Nevertheless, none of the OCT proteins can transport natural nucleosides. The possibility of ENT4 playing a role in adenosine transport was raised by Barnes et al. (2006) several years ago. These authors demonstrated that serotonin transport via ENT4 was not pH-dependent, whereas adenosine transport was. Apparent Km values for adenosine at acidic pH (5.5) were in the high micromolar range but still were considered to be compatible with ENT4 being an adenosine transporter protein in physiological conditions associated with acidosis.

In summary, we have briefly dissected and discussed the basic biochemical principles and events governing adenosine transport into cells, by highlighting which are the best transporter candidates to regulate extracellular adenosine levels, and therefore, adenosine-mediated purinergic signaling.

## Adenosine Transporters and Purinergic Signaling

Once the plasma membrane transporters likely to be implicated in the regulation of adenosine levels have been identified, we will discuss what is the physiological evidence supporting a functional link between a particular transporter subtype and purinergic regulation.

Several experimental approaches have been used in this regard. NT transporter pharmacology is still poorly developed and no subtype-specific inhibitors are available for CNT proteins, although this is not the case for ENTs. Indeed, high-affinity inhibition of ENT1 by NMBPR has proven very helpful. In fact, the determination of NMBPR-specific binding sites has been used by different authors to quantify ENT1 expression at the plasma membrane, even long before ENT1 was identified at the molecular level (Pickard et al., 1973; Dahlig-Harley et al., 1981; Marangos et al., 1982). Besides the pharmacological approach, functional genomics is also available for ENT1, since Choi colleagues reported the first NT-subtype knock out mouse model (Choi et al., 2004; Oliveros et al., 2017). Probably because of these circumstances, we can say that ENT1 is the most studied transporter among the two families (*SLC28* and *SLC29*), with plenty of literature showing a link between ENT1 function and purinergic regulation. Another experimental approach suitable for the analysis of adenosine transporters as players in the purinome, comes from the evidence that selected NT subtypes (including ENT1) are under purinergic control. This means that their function is regulated by P1 (but probably also by P2) type receptors. In the classical set up of purinome function one would envisage NT proteins being stimulated by adenosine acting on P1 receptors, thereby promoting extracellular adenosine removal and ending the purinergic signaling. Moreover, changes in the expression of particular NT subtypes in physiological and pathophysiological conditions known to be associated with increased adenosine levels, further support the role particular NT proteins might play in purinergic regulation.

### ENT Proteins

ENT1 expression in the rat and human brain has been mapped by different means (i.e., NBMPR binding, mRNA *in situ* hybridization and others). ENT1 shows broad cellular and regional distribution and its role in adenosine signaling is relatively well-understood (Parkinson et al., 2011). Adenosine is known to be neuroprotective in various pathological conditions such as stroke (Cunha, 2016). This is the reason why physiological mechanisms governing adenosine extracellular levels have been comprehensively studied. A probable dual role of ENT proteins either as influx or efflux transporters has been reported in the CNS. Indeed, rat cortical neurons when cultured alone are able to release adenosine after *N*-methyl-D-aspartate (NMDA) stimulation, whereas the NMDA-triggered increase in extracellular adenosine concentration appears to be related to nucleotide degradation when neurons are co-cultured with astrocytes (Zamzow et al., 2008). In rat hippocampal slices it has been shown that ATP is able to promote adenosine release via ENT-type proteins, which in turn might activate A2A receptors (Almeida et al., 2003). Subsequently, A2A activation might promote adenosine uptake, as shown in hippocampal synaptosomes (Pinto-Duarte et al., 2005). In fact, adenosine uptake via ENT-type transporters appears to reduce extracellular adenosine levels in hypoxia which suggests that ENT proteins and probably ENT1 in particular might be suitable targets for the treatment of cerebral ischemia (Zhang et al., 2011). Interestingly, adenosine in the brain has also been related to addictive behaviors, among them alcohol addiction. It has been known for a long time that ethanol increases extracellular adenosine by inhibiting in a somehow selective manner ENT1 function (Nagy et al., 1990). Nevertheless the most conclusive evidence supporting this pharmacological effect comes from functional genomics. The ENT1 knock out mouse model shows reduced acute responses to ethanol intake and increased addiction to alcohol (Choi et al., 2004). This animal model has also been useful in the understanding of ENT1-related adenosine signaling in other organs. In fact, ENT1-null mice show increased adenosine plasma levels and are cardioprotected (Rose et al., 2010, 2011). Similarly, ENT1 appears to be implicated in adenosine-related protection in the liver during ischemia and reperfusion (Zimmerman et al., 2013).

Moreover, adenosine contributes to chronic kidney disease, particularly in diabetes. Increased adenosine signaling via A2B receptors has been reported to be involved in diabetic glomerulopathy (Cárdenas et al., 2013), and increased adenosine levels in insulin-deficient states have been associated with down-regulation of ENT2 transport function in podocytes (Alarcón et al., 2017). In a complementary manner, in proximal tubule cells, decreased ENT1 function has also been related to fibrosis in diabetic nephropathy (Kretschmar et al., 2016). In fact, ENT1 null mouse shows a spontaneous tendency to develop renal fibrosis whereas ENT1 silencing in human kidney epithelial (HK) cells results in the promotion of epithelial-to-mesenchymal transition (EMT) (Guillén-Gómez et al., 2012). Promotion of EMT in HK2 cells can be mimicked by TFG-β1, whereas adenosine itself mediates TFG-β1 release from glomeruli of diabetic rats via A2B receptor activation (Roa et al., 2009).

The involvement of ENT proteins in the regulation of adenosine tone in vascular endothelium has been comprehensively studied using Human Umbilical Vein Endothelial Cells (HUVECs) and Placenta Microvascular Endothelial Cells (PMECs) as experimental models (review in Sobrevia et al., 2011; Pardo et al., 2013). As in other cell types, it has been shown that control of extracellular adenosine levels via P1 receptors also involves hENT modulation, in particular the hENT1 and hENT2 subtypes (Escudero et al., 2008; Pardo et al., 2013). In some cases, opposite effects on each transporter protein have been reported, thereby suggesting either some sort of physiological compensation or an hENT-subtype specific effect impacting on the ability of removing from the extracellular milieu not only adenosine but also some of its catabolites. hENT2 is indeed a suitable hypoxanthine transporter. Interestingly, we have recently shown that hENT1 and hENT2 can form oligomers hENT1-hENT1, hENT2-hENT2, but also hENT1-hENT2, with multiple functional consequences (Grañé-Boladeras et al., 2002).

Overall, it is within this framework that the pharmacological use of ENT inhibitors such as dipyridamole and dilazep can be understood (Figueredo et al., 1999). Nevertheless, at least for cardioprotection, it has recently been argued that ENT4 could become a better target than ENT1, because of the more restricted tissue expression pattern of the former ENT subtype (Yang and Leung, 2015). As discussed above, ENT4 was shown to be a suitable cardiac adenosine transporter at acidic pH (Barnes et al., 2006). Then, the idea these authors discuss is that most cell types may rely upon ENT1 for nucleoside salvage purposes, thereby making any ENT1-targeting drug more likely to present adverse effects than newly developed molecules targeting ENT4.

### CNT Proteins

As discussed above, hCNT2 and hCNT3 should be, by far, the best candidates for efficient removal of adenosine from the extracellular milieu. This statement is based upon their apparent high affinity for adenosine and for its concentrative capacity which is even 10 fold higher for hCNT3 than hCNT2. Nevertheless, hCNT pharmacology is very poor, even though some hCNT subtype specific inhibitors have been recently proposed (Kumar Deokar et al., 2017). In this regard the structural modeling of human CNT subtypes (Arimany-Nardi et al., 2017; Kumar Deokar et al., 2017; Latek, 2017; Mulinta et al., 2017) based upon the crystal structure of the *Vibrio cholerae* CNT ortholog (Johnson et al., 2012) might be particularly useful for future specific inhibitor design. This has been a major bottleneck to study the probable impact of acute CNT pharmacological inhibition on adenosine signaling. Nevertheless there is solid experimental evidence showing that CNT2 and CNT3 are under purinergic regulation, which suggests they contribute, as ENTs, to modulate extracellular adenosine levels and P1 signaling.

In liver parenchymal cells CNT2 is expressed at the basolateral (sinusoidal) and apical (canalicular) plasma membranes (Duflot et al., 2002; Govindarajan et al., 2008). In rat primary hepatocytes and hepatocarcinoma FAO cells the activity of this transporter protein is under purinergic regulation via A1 receptors (Duflot et al., 2004). This effect is relatively rapid (peaking between 5 and 10 min after agonist addition) and consistent with increased transport capacity (Vmax effect). Interestingly the magnitude of the effect (transport fold-induction) is dependent upon glucose concentration, being lower at high glucose (10 vs. 5 mM glucose). Indeed, CNT2 up-regulation could be blocked by inhibitors of KATP channels and mimicked by openers, which establishes a putative link between energy metabolism and purinergic regulation of CNT2. All the protein machinery likely to be implicated in this phenomenon, this is the KATP channel subunits Kir6.1, Kir6.2, SUR2A, SUR2B, as well as the transporter itself and A1 receptors were shown to co-localize in FAO cells. The physiological impact of the reduction of adenosine removal capacity triggered by high-glucose is not clear, although decreased hENT1 function and expression have been reported in HUVEC from diabetic patients and shown to be mimicked by high glucose in the culture medium (Sobrevia et al., 2011). It is interesting to keep in mind that extracellular adenosine has been reported to be able to modulate the AMP-dependent kinase AMPK, by a mechanism which depends on transporter function, also involving CNT2 in some cell types (Aymerich et al., 2006). Overall, CNT2 appears to be a suitable candidate to modulate purinergic signaling in hepatocytes, particularly considering that hCNT1 is not an adenosine transporter and hCNT3 expression in hepatocytes appears to be negligible. Interestingly, CNT2 function has recently been identified in primary rat bile duct epithelial cells where it is similarly found in both plasma membrane domains, apical and basolateral of cholangiocytes (Godoy et al., 2014). Luminal ATP, via P2Y receptors, down-regulates apical (lumen-facing) CNT2 activity by a Ca^++^-dependent mechanism. Cholangiocytes also express CNT3 and its apical function is similarly down-regulated by nucleotides, such as ATP. However, A2A agonists (i.e., adenosine), acting from the luminal side, specifically activate apical CNT3, without modifying CNT2 function. CNT3 activation is consistent with transporter trafficking from intracellular vesicles to the plasma membrane. In practice this means that CNT2 and probably to more extent CNT3 are contributing to end up the purinergic regulation of bile flow by removing adenosine from the bile canaliculus. It makes sense that the adenosine precursor ATP reduces adenosine removal capacity by inhibiting both CNT2 and CNT3, whereas the differential regulation of both transporters by adenosine acting on A2A receptors may reflect basal (CNT2 and CNT3) and adenosine-induced (CNT3) capacity for its own removal.

CNT2, in parallel with ENT1, has also been mapped in the adult rat brain by *in situ* hybridization (Guillén-Gómez et al., 2004). Indeed, CNT2 is broadly distributed in the CNS with significant overlapping with ENT1. More recently, others have identified the CNT2 protein in plasma and vesicle membranes isolated from rat striatum (Melani et al., 2012). The possibility of CNT2 also playing a role in adenosine signaling in the brain is also supported by the evidence that its activity can be up-regulated by P1 receptor activation in differentiated neural PC12 cells (most probably A1 and A2A) (Medina-Pulido et al., 2013). CNT2 activation is relatively rapid, as in hepatocytes, peaking 15 min after P1 agonist addition. Interestingly, caffeine has been reported to inhibit CNT2 function with an apparent Ki value of 103 μM (Lang et al., 2004). Although this concentration might significantly exceed the one found in blood after coffee ingestion, it could still be relevant in heavy coffee drinkers (Nehlig and Debry, 1994). Taking together these observations suggest ENT1 may not be the only player regulating adenosine signaling in the brain, which in fact is consistent with the relatively mild phenotype of the ENT1 null mice.

Last, but not least, several physiological and pathophysiological observations also support a role for hCNT2 and hCNT3 as proteins relevant to purinergic signaling. The three hCNT genes are expressed in the nephron and accurate anatomic analysis of their distribution along it reveals a longitudinal pattern of expression consistent with nucleoside renal tubule reabsorption but also with adenosine-mediated tubulo-glomerular feedback regulation (Schnermann, 2015). Indeed, the three transporter proteins are expressed in the proximal convoluted tubule (PCT), where most nutrient reabsorption (glucose, amino acids) take place, but only the two adenosine transporters (CNT2 and CNT3), not CNT1, are also expressed in very specific distal segments of the nephron, the cortical collecting duct (CCD) (CNT3) and the outer medullary collecting duct (OMCD) (CNT2). This anatomical distribution is more consistent with adenosine signaling than with nucleoside reabsorption. Distribution of CNTs along the gastrointestinal tract (Pastor-Anglada et al., 2018) also points to this dual role of adenosine transporters. In fact, the CNT2 encoding gene (*SLC28A2*) is, by far, the one which is down-regulated the most (sevenfold) in inflamed ileon mucosa from Crohn’s patients (Pérez-Torras et al., 2016). Even though the impact of inflammation is broad and down-regulates a broad cohort of genes, those associated with the purinome (transporters, receptors, and ectonucleotidases) are greatly affected.

In the rat brain, CNT2 is also down-regulated in situations known to be associated with increased adenosine concentrations. CNT2 mRNA levels are decreased in cortical samples from sleep-deprived rats, whereas ENT1 mRNA is not affected at all under the same circumstances (Guillén-Gómez et al., 2004). CNT2-related mRNA tends to recover when animals are allowed to sleep. On the other hand, experimental ischemia *in vivo*, induced in rats by intraluminal middle cerebral arterial occlusion, also regulates nucleoside transporter encoding genes (Medina-Pulido et al., 2013). In this study, transcripts of both ENTs (ENT1 and ENT2) and the three CNT members were quantified in the ipsilateral cortex (infarcted) and compared to the contralateral cortex as its own control. Indeed, the mRNA levels of hENT1, hCNT2, but also hCNT3 (poorly studied in the brain), the three transporters more likely to modulate adenosine tone, were decreased in the infarcted tissue with no changes observed for hCNT1 and hENT2.

## Concluding Remarks and Future Perspectives

Nucleoside transport by each particular nucleoside protein subtype cannot be measured directly, thereby resulting in some experimental variability likely to impact on the determination of adenosine affinity constants. Despite this limitation, we can conclude that adenosine transport mechanisms across the plasma membrane are well-understood with the only exception of ENT4, for which a clear role in the regulation of adenosine tone in some tissues (i.e., heart) still awaits clarification. Nevertheless, in general trends, who is who in adenosine transport is well-known.

Less clear is how each transporter subtype contributes to modulate adenosine levels, an issue of particular interest considering most cells show some apparent redundancy in the expression of adenosine transporters. ENT1 is by far the most studied adenosine transporter. This may be explained, as discussed above, not necessarily because of its ubiquitous expression, but because pharmacological tools and functional genomics have provided better chances to study it than for the other adenosine transporters. Nevertheless, the contribution of the other subtypes, particularly CNTs (CNT2 and CNT3) should not be ruled out and deserves further investigation.

Although CNT and ENT subtype expression appears to be polarized in (re)absorptive epithelia, thus allowing vectorial flux of substrates, in other epithelial cell types CNT and ENT proteins appear to be located in both poles (apical and basolateral), thereby anticipating other roles beyond absorption (Pastor-Anglada et al., 2018). Moreover, non-epithelial cells, such as adipocytes (Guallar et al., 2007) and immune system cells (Soler et al., 1998, 2001; Minuesa et al., 2009, 2011) also express ENT and CNT proteins. Whether particular subtypes are under purinergic regulation in these cells and tissues has not been properly addressed until now. Last, but not least, there is also the possibility of local regulation of selected adenosine transporters even at the single cell level, thereby providing some sort of compartmental regulation of biological functions. In this regard, ENT and CNT protein interactomics might help to unveil novel regulatory events likely to facilitate the fine tuning of purinergic regulation.

## Author Contributions

MP-A conceived the review and wrote the first draft of the manuscript. SP-T critiqued and revised the manuscript. SP-T and MP-A read the final version of the manuscript and approved it for submission.

## Conflict of Interest Statement

The authors declare that the research was conducted in the absence of any commercial or financial relationships that could be construed as a potential conflict of interest.

## References

[B1] Alarcón S. Garrido W. Cappelli C. Suárez R. Oyarzún C. Quezada C. (2017). Deficient insulin-mediated upregulation of the equilibrative nucleoside transporter 2 contributes to chronically increased adenosine in diabetic glomerulopathy. *Sci. Rep.* 7:9439. 10.1038/s41598-017-09783-0 28842605 PMC5572683

[B2] Almeida T. Rodrigues R. J. de Mendonça A. Ribeiro J. A. Cunha R. A. (2003). Purinergic P2 receptors trigger adenosine release leading to adenosine A2A receptor activation and facilitation of long-term potentiation in rat hippocampal slices. *Neuroscience* 122 111–121. 10.1016/S0306-4522(03)00523-2 14596853

[B3] Arimany-Nardi C. Claudio-Montero A. Viel-Oliva A. Schmidtke P. Estarellas C. Barril X. (2017). Identification and characterization of a secondary sodium-binding site and the main selectivity determinants in the human concentrative nucleoside transporter 3. *Mol. Pharm.* 14 1980–1987. 10.1021/acs.molpharmaceut.7b00085 28441873

[B4] Aymerich I. Foufelle F. Ferré P. Casado F. J. Pastor-Anglada M. (2006). Extracellular adenosine activates AMP-dependent protein kinase (AMPK). *J. Cell Sci.* 119(Pt 8), 1612–1621. 10.1242/jcs.02865 16569664

[B5] Baldwin S. A. Yao S. Y. Hyde R. J. Ng A. M. Foppolo S. Barnes K. (2005). Functional characterization of novel human and mouse equilibrative nucleoside transporters (hENT3 and mENT3) located in intracellular membranes. *J. Biol. Chem.* 280 15880–15887. 10.1074/jbc.M414337200 15701636

[B6] Barnes K. Dobrzynski H. Foppolo S. Beal P. R. Ismat F. Scullion E. R. (2006). Distribution and functional characterization of equilibrative nucleoside transporter-4, a novel cardiac adenosine transporter activated at acidic pH. *Circ. Res.* 99 510–519. 10.1161/01.RES.0000238359.18495.42 16873718

[B7] Blay J. White T. D. Hoskin D. W. (1997). The extracellular fluid of solid carcinomas contains immunosuppressive concentrations of adenosine. *Cancer Res.* 57 2602–2605. 9205063

[B8] Boleti H. Coe I. R. Baldwin S. A. Young J. D. Cass C. E. (1997). Molecular identification of the equilibrative NBMPR-sensitive (es) nucleoside transporter and demonstration of an equilibrative NBMPR-insensitive (ei) transport activity in human erythroleukemia (K562) cells. *Neuropharmacology* 36 1167–1179. 10.1016/S0028-3908(97)00136-6 9364472

[B9] Burnstock G. (2017). Purinergic signalling: therapeutic developments. *Front. Pharmacol.* 8:661 10.3389/fphar.2017.00661 PMC5622197 28993732

[B10] Cárdenas A. Toledo C. Oyarzún C. Sepúlveda A. Quezada C. Guillén-Gómez E. (2013). Adenosine A(2B) receptor-mediated VEGF induction promotes diabetic glomerulopathy. *Lab. Invest.* 93 135–144. 10.1038/labinvest.2012.143 23069939

[B11] Casanello P. Torres A. Sanhueza F. González M. Farías M. Gallardo V. (2005). Equilibrative nucleoside transporter 1 expression is downregulated by hypoxia in human umbilical vein endothelium. *Circ. Res.* 97 16–24. 10.1161/01.RES.0000172568.49367.f8 15933265

[B12] Celis N. Araos J. Sanhueza C. Toledo F. Beltrán A. R. Pardo F. (2017). Intracellular acidification increases adenosine transport in human umbilical vein endothelial cells. *Placenta* 51 10–17. 10.1016/j.placenta.2017.01.120 28292464

[B13] Che M. Ortiz D. F. Arias I. M. (1995). Primary structure and functional expression of a cDNA encoding the bile canalicular, purine-specific Na(+)-nucleoside cotransporter. *J. Biol. Chem.* 270 13596–13599. 10.1074/jbc.270.23.13596 7775409

[B14] Choi D. S. Cascini M. G. Mailliard W. Young H. Paredes P. McMahon T. (2004). The type 1 equilibrative nucleoside transporter regulates ethanol intoxication and preference. *Nat. Neurosci.* 7 855–861. 10.1038/nn1288 15258586

[B15] Cunha R. A. (2016). How does adenosine control neuronal dysfunction and neurodegeneration? *J. Neurochem.* 139 1019–1055. 10.1111/jnc.13724 27365148

[B16] Dahlig-Harley E. Eilam Y. Paterson A. R. Cass C. E. (1981). Binding of nitrobenzylthioinosine to high-affinity sites on the nucleoside-transport mechanism of HeLa cells. *Biochem. J.* 200 295–305. 10.1042/bj2000295 6280683 PMC1163535

[B17] Damaraju S. Zhang J. Visser F. Tackaberry T. Dufour J. Smith K. M. (2005). Identification and functional characterization of variants in human concentrative nucleoside transporter 3, hCNT3 (SLC28A3), arising from single nucleotide polymorphisms in coding regions of the hCNT3 gene. *Pharmacogenet. Genomics* 15 173–182. 10.1097/01213011-200503000-00006 15861042

[B18] de Andrade Mello P. Coutinho-Silva R. Savio L. E. B. (2017). Multifaceted effects of extracellular adenosine triphosphate and adenosine in the tumor-host interaction and therapeutic perspectives. *Front. Immunol.* 8:1526. 10.3389/fimmu.2017.01526 29184552 PMC5694450

[B19] Di Virgilio F. Adinolfi E. (2017). Extracellular purines, purinergic receptors and tumor growth. *Oncogene* 36 293–303. 10.1038/onc.2016.206 27321181 PMC5269532

[B20] Dos Santos-Rodrigues A. Grane-Boladeras N. Bicket A. Coe I. R. (2014). Nucleoside transporters in the purinome. *Neurochem. Int.* 73 229–237. 10.1016/j.neuint.2014.03.014 24704797

[B21] Duflot S. Calvo M. Casado F. J. Enrich C. Pastor-Anglada M. (2002). Concentrative nucleoside transporter (rCNT1) is targeted to the apical membrane through the hepatic transcytotic pathway. *Exp. Cell Res.* 281 77–85. 10.1006/excr.2002.5641 12441131

[B22] Duflot S. Riera B. Fernández-Veledo S. Casadó V. Norman R. I. Casado F. J. (2004). ATP-sensitive K(+) channels regulate the concentrative adenosine transporter CNT2 following activation by A(1) adenosine receptors. *Mol. Cell Biol.* 24 2710–2719. 10.1128/MCB.24.7.2710-2719.2004 15024061 PMC371120

[B23] Engel K. Zhou M. Wang J. (2004). Identification and characterization of a novel monoamine transporter in the human brain. *J. Biol. Chem.* 279 50042–50049. 10.1074/jbc.M407913200 15448143

[B24] Errasti-Murugarren E. Cano-Soldado P. Pastor-Anglada M. Casado F. J. (2008). Functional characterization of a nucleoside-derived drug transporter variant (hCNT3C602R) showing altered sodium-binding capacity. *Mol. Pharmacol.* 73 379–386. 10.1124/mol.107.041848 17993510

[B25] Errasti-Murugarren E. Molina-Arcas M. Casado F. J. Pastor-Anglada M. (2009). A splice variant of the SLC28A3 gene encodes a novel human concentrative nucleoside transporter-3 (hCNT3) protein localized in the endoplasmic reticulum. *FASEB J.* 23 172–182. 10.1096/fj.08-113902 18827020

[B26] Errasti-Murugarren E. Molina-Arcas M. Casado F. J. Pastor-Anglada M. (2010). The human concentrative nucleoside transporter-3 C602R variant shows impaired sorting to lipid rafts and altered specificity for nucleoside-derived drugs. *Mol. Pharmacol.* 78 157–165. 10.1124/mol.110.063552 20421346

[B27] Escudero C. Casanello P. Sobrevia L. (2008). Human equilibrative nucleoside transporters 1 and 2 may be differentially modulated by A2B adenosine receptors in placenta microvascular endothelial cells from pre-eclampsia. *Placenta* 29 816–825. 10.1016/j.placenta.2008.06.014 18703227

[B28] Espinoza J. Espinoza A. F. Power G. G. (2011). High fetal plasma adenosine concentration: a role for the fetus in preeclampsia? *Am. J. Obstet. Gynecol.* 485 e424–e487. 10.1016/j.ajog.2011.06.034 21855848

[B29] Fenton R. A. Dobson J. G. Jr. (1992). Fluorometric quantitation of adenosine concentration in small samples of extracellular fluid. *Anal. Biochem.* 207 134–141. 10.1016/0003-2697(92)90514-8 1489086

[B30] Figueredo V. M. Diamond I. Zhou H. Z. Albert Camacho S. (1999). Chronic dipyridamole therapy produces sustained protection against cardiac ischemia-reperfusion injury. *Am. J. Physiol.* 277(5 Pt 2), H2091–H2097. 10.1152/ajpheart.1999.277.5.H2091 10564165

[B31] Fredholm B. B. IJzerman A. P. Jacobson K. A. Linden J. Müller C. E. (2011). International Union of Basic and Clinical Pharmacology. LXXXI. Nomenclature and classification of adenosine receptors–an update. *Pharmacol. Rev.* 63 1–34. 10.1124/pr.110.003285 21303899 PMC3061413

[B32] Godoy V. Banales J. M. Medina J. F. Pastor-Anglada M. (2014). Functional crosstalk between the adenosine transporter CNT3 and purinergic receptors in the biliary epithelia. *J. Hepatol.* 61 1337–1343. 10.1016/j.jhep.2014.06.036 25034758

[B33] Gorraitz E. Pastor-Anglada M. Lostao M. P. (2010). Effects of Na+ and H+ on steady-state and presteady-state currents of the human concentrative nucleoside transporter 3 (hCNT3). *Pflugers Arch.* 460 617–632. 10.1007/s00424-010-0846-9 20495821

[B34] Govindarajan R. Endres C. J. Whittington D. LeCluyse E. Pastor-Anglada M. Tse C. M. (2008). Expression and hepatobiliary transport characteristics of the concentrative and equilibrative nucleoside transporters in sandwich-cultured human hepatocytes. *Am. J. Physiol. Gastrointest. Liver Physiol.* 295 G570–G580. 10.1152/ajpgi.00542.2007 18635603 PMC2536788

[B35] Grañé-Boladeras N. Spring C. M. Hanna W. J. Pastor-Anglada M. Coe I. R. (2016). Novel nuclear hENT2 isoforms regulate cell cycle progression via controlling nucleoside transport and nuclear reservoir. *Cell Mol. Life Sci.* 73 4559–4575. 10.1007/s00018-016-2288-9 27271752 PMC11108336

[B36] Grañé-Boladeras N. Williams D. McKenzie T. Naydenova Z. (2002). Oligomerisation of Equilibrative Nucleoside Transporters: a novel regulatory and functional mechanism involving PKC and PP1. *FASEB J.* 517 201–205. 10.1096/fj.201800440RR 30521377

[B37] Guallar J. P. Cano-Soldado P. Aymerich I. Domingo J. C. Alegre M. Domingo P. (2007). Altered expression of nucleoside transporter genes (SLC28 and SLC29) in adipose tissue from HIV-1-infected patients. *Antivir. Ther.* 12 853–863. 17926640

[B38] Guillén-Gómez E. Calbet M. Casado J. de Lecea L. Soriano E. Pastor-Anglada M. (2004). Distribution of CNT2 and ENT1 transcripts in rat brain: selective decrease of CNT2 mRNA in the cerebral cortex of sleep-deprived rats. *J. Neurochem.* 90 883–893. 10.1111/j.1471-4159.2004.02545.x 15287894

[B39] Guillén-Gómez E. Pinilla-Macua I. Pérez-Torras S. Choi D. S. Arce Y. Ballarín J. A. (2012). New role of the human equilibrative nucleoside transporter 1 (hENT1) in epithelial-to-mesenchymal transition in renal tubular cells. *J. Cell Physiol.* 227 1521–1528. 10.1002/jcp.22869 21678404

[B40] Hsu C. L. Lin W. Seshasayee D. Chen Y. H. Ding X. Lin Z. (2012). Equilibrative nucleoside transporter 3 deficiency perturbs lysosome function and macrophage homeostasis. *Science* 335 89–92. 10.1126/science.1213682 22174130

[B41] Huang Q. Q. Yao S. Y. Ritzel M. W. Paterson A. R. Cass C. E. Young J. D. (1994). Cloning and functional expression of a complementary DNA encoding a mammalian nucleoside transport protein. *J. Biol. Chem.* 269 17757–17760. 8027026

[B42] Johnson Z. L. Cheong C. G. Lee S. Y. (2012). Crystal structure of a concentrative nucleoside transporter from *Vibrio cholerae* at 2.4 A. *Nature* 483 489–493. 10.1038/nature10882 22407322 PMC3310960

[B43] Kang N. Jun A. H. Bhutia Y. D. Kannan N. Unadkat J. D. Govindarajan R. (2010). Human equilibrative nucleoside transporter-3 (hENT3) spectrum disorder mutations impair nucleoside transport, protein localization, and stability. *J. Biol. Chem.* 285 28343–28352. 10.1074/jbc.M110.109199 20595384 PMC2934698

[B44] Kretschmar C. Oyarzún C. Villablanca C. Jaramillo C. Alarcón S. Perez G. (2016). Reduced adenosine uptake and its contribution to signaling that mediates profibrotic activation in renal tubular epithelial cells: implication in diabetic nephropathy. *PLoS One* 11:e0147430. 10.1371/journal.pone.0147430 26808537 PMC4726618

[B45] Kumar Deokar H. Barch H. P. Barch H. P. Buolamwini J. K. (2017). Homology modeling of human concentrative nucleoside transporters (hCNTs) and validation by virtual screening and experimental testing to identify novel hCNT1 Inhibitors. *Drug Des.* 6:146. 10.4172/2169-0138.1000146 29167753 PMC5695676

[B46] Lang T. T. Young J. D. Cass C. E. (2004). Interactions of nucleoside analogs, caffeine, and nicotine with human concentrative nucleoside transporters 1 and 2 stably produced in a transport-defective human cell line. *Mol. Pharmacol.* 65 925–933. 10.1124/mol.65.4.925 15044622

[B47] Larráyoz I. M. Casado F. J. Pastor-Anglada M. Lostao M. P. (2004). Electrophysiological characterization of the human Na(+)/nucleoside cotransporter 1 (hCNT1) and role of adenosine on hCNT1 function. *J. Biol. Chem.* 279 8999–9007. 10.1074/jbc.M311940200 14701834

[B48] Larráyoz I. M. Fernández-Nistal A. Garcés A. Gorraitz E. Lostao M. P. (2006). Characterization of the rat Na+/nucleoside cotransporter 2 and transport of nucleoside-derived drugs using electrophysiological methods. *Am. J. Physiol. Cell Physiol.* 291 C1395–C1404. 10.1152/ajpcell.00110.2006 16837649

[B49] Latek D. (2017). Rosetta Broker for membrane protein structure prediction: concentrative nucleoside transporter 3 and corticotropin-releasing factor receptor 1 test cases. *BMC Struct. Biol.* 17:8. 10.1186/s12900-017-0078-8 28774292 PMC5543540

[B50] Li J. Y. Boado R. J. Pardridge W. M. (2001). Differential kinetics of transport of 2′,3′-dideoxyinosine and adenosine via concentrative Na+ nucleoside transporter CNT2 cloned from rat blood-brain barrier. *J. Pharmacol. Exp. Ther.* 299 735–740. 11602688

[B51] Lostao M. P. Mata J. F. Larrayoz I. M. Inzillo S. M. Casado F. J. Pastor-Anglada M. (2000). Electrogenic uptake of nucleosides and nucleoside-derived drugs by the human nucleoside transporter 1 (hCNT1) expressed in Xenopus laevis oocytes. *FEBS Lett.* 481 137–140. 10.1016/S0014-5793(00)01983-9 10996312

[B52] Mackey J. R. Mani R. S. Selner M. Mowles D. Young J. D. Belt J. A. (1998). Functional nucleoside transporters are required for gemcitabine influx and manifestation of toxicity in cancer cell lines. *Cancer Res.* 58 4349–4357. 9766663

[B53] Marangos P. J. Patel J. Clark-Rosenberg R. Martino A. M. (1982). [3H]nitrobenzylthioinosine binding as a probe for the study of adenosine uptake sites in brain. *J. Neurochem.* 39 184–191. 10.1111/j.1471-4159.1982.tb04717.x 7086410

[B54] Medina-Pulido L. Molina-Arcas M. Justicia C. Soriano E. Burgaya F. Planas A. M. (2013). Hypoxia and P1 receptor activation regulate the high-affinity concentrative adenosine transporter CNT2 in differentiated neuronal PC12 cells. *Biochem. J.* 454 437–445. 10.1042/BJ20130231 23819782

[B55] Melani A. Corti F. Stephan H. Müller C. E. Donati C. Bruni P. (2012). Ecto-ATPase inhibition: ATP and adenosine release under physiological and ischemic in vivo conditions in the rat striatum. *Exp. Neurol.* 233 193–204. 10.1016/j.expneurol.2011.09.036 22001157

[B56] Minuesa G. Huber-Ruano I. Pastor-Anglada M. Koepsell H. Clotet B. Martinez-Picado J. (2011). Drug uptake transporters in antiretroviral therapy. *Pharmacol. Ther.* 132 268–279. 10.1016/j.pharmthera.2011.06.007 21816170

[B57] Minuesa G. Volk C. Molina-Arcas M. Gorboulev V. Erkizia I. Arndt P. (2009). Transport of lamivudine [(-)-beta-L-2′,3′-dideoxy-3′-thiacytidine] and high-affinity interaction of nucleoside reverse transcriptase inhibitors with human organic cation transporters 1, 2, and 3. *J. Pharmacol. Exp. Ther.* 329 252–261. 10.1124/jpet.108.146225 19141712

[B58] Mulinta R. Yao S. Y. M. Ng A. M. L. Cass C. E. Young J. D. (2017). Substituted cysteine accessibility method (SCAM) analysis of the transport domain of human concentrative nucleoside transporter 3 (hCNT3) and other family members reveals features of structural and functional importance. *J. Biol. Chem.* 292 9505–9522. 10.1074/jbc.M116.743997 28385889 PMC5465479

[B59] Muñoz G. San Martín R. Farías M. Cea L. Vecchiola A. Casanello P. (2006). Insulin restores glucose inhibition of adenosine transport by increasing the expression and activity of the equilibrative nucleoside transporter 2 in human umbilical vein endothelium. *J. Cell Physiol.* 209 826–835. 10.1002/jcp.20769 16924660

[B60] Nagy L. E. Diamond I. Casso D. J. Franklin C. Gordon A. S. (1990). Ethanol increases extracellular adenosine by inhibiting adenosine uptake via the nucleoside transporter. *J. Biol. Chem.* 265 1946–1951. 2298733

[B61] Nehlig A. Debry G. (1994). Potential teratogenic and neurodevelopmental consequences of coffee and caffeine exposure: a review on human and animal data. *Neurotoxicol. Teratol.* 16 531–543. 10.1016/0892-0362(94)90032-9 7862054

[B62] Nguyen M. D. Ross A. E. Ryals M. Lee S. T. Venton B. J. (2015). Clearance of rapid adenosine release is regulated by nucleoside transporters and metabolism. *Pharmacol. Res. Perspect.* 3:e00189. 10.1002/prp2.189 27022463 PMC4777247

[B63] Oliveros A. Starski P. Lindberg D. Choi S. Heppelmann C. J. Dasari S. (2017). Label-Free neuroproteomics of the hippocampal-accumbal circuit reveals deficits in neurotransmitter and neuropeptide signaling in mice lacking ethanol-sensitive adenosine transporter. *J. Proteome Res.* 16 1445–1459. 10.1021/acs.jproteome.6b00830 27998058 PMC5384880

[B64] Pardo F. Arroyo P. Salomón C. Westermeier F. Salsoso R. Sáez T. (2013). Role of equilibrative adenosine transporters and adenosine receptors as modulators of the human placental endothelium in gestational diabetes mellitus. *Placenta* 34 1121–1127. 10.1016/j.placenta.2013.09.007 24119573

[B65] Parkinson F. E. Damaraju V. L. Graham K. Yao S. Y. Baldwin S. A. Cass C. E. (2011). Molecular biology of nucleoside transporters and their distributions and functions in the brain. *Curr. Top. Med. Chem.* 11 948–972. 10.2174/156802611795347582 21401500

[B66] Pastor-Anglada M. Urtasun N. Pérez-Torras S. (2018). Intestinal nucleoside transporters: function, expression and regulation. *Comp. Physiol.* (in press). 10.1002/cphy.c170039 29978890

[B67] Pérez-Torras S. Iglesias I. Llopis M. Lozano J. J. Antolín M. Guarner F. (2016). Transportome profiling identifies profound alterations in crohn’s disease partially restored by commensal bacteria. *J. Crohns Colitis* 10 850–859. 10.1093/ecco-jcc/jjw042 26874350

[B68] Pickard M. A. Brown R. R. Paul B. Paterson A. R. (1973). Binding of the nucleoside transport inhibitor 4-nitrobenzylthioinosine to erythrocyte membranes. *Can. J. Biochem.* 51 666–672. 10.1139/o73-083 4706840

[B69] Pinto-Duarte A. Coelho J. E. Cunha R. A. Ribeiro J. A. Sebastião A. M. (2005). Adenosine A2A receptors control the extracellular levels of adenosine through modulation of nucleoside transporters activity in the rat hippocampus. *J. Neurochem.* 93 595–604. 10.1111/j.1471-4159.2005.03071.x 15836618

[B70] Rahman M. F. Askwith C. Govindarajan R. (2017). Molecular determinants of acidic pH-dependent transport of human equilibrative nucleoside transporter 3. *J. Biol. Chem.* 292 14775–14785. 10.1074/jbc.M117.787952 28729424 PMC5592659

[B71] Ritzel M. W. Ng A. M. Yao S. Y. Graham K. Loewen S. K. Smith K. M. (2001). Molecular identification and characterization of novel human and mouse concentrative Na+-nucleoside cotransporter proteins (hCNT3 and mCNT3) broadly selective for purine and pyrimidine nucleosides (system cib). *J. Biol. Chem.* 276 2914–2927. 10.1074/jbc.M007746200 11032837

[B72] Roa H. Gajardo C. Troncoso E. Fuentealba V. Escudero C. Yáñez A. (2009). Adenosine mediates transforming growth factor-beta 1 release in kidney glomeruli of diabetic rats. *FEBS Lett.* 583 3192–3198. 10.1016/j.febslet.2009.09.003 19737558

[B73] Rose J. B. Naydenova Z. Bang A. Eguchi M. Sweeney G. Choi D. S. (2010). Equilibrative nucleoside transporter 1 plays an essential role in cardioprotection. *Am. J. Physiol. Heart Circ. Physiol.* 298 H771–H777. 10.1152/ajpheart.00711.2009 20035027 PMC3774072

[B74] Rose J. B. Naydenova Z. Bang A. Ramadan A. Klawitter J. Schram K. (2011). Absence of equilibrative nucleoside transporter 1 in ENT1 knockout mice leads to altered nucleoside levels following hypoxic challenge. *Life Sci.* 89 621–630. 10.1016/j.lfs.2011.08.007 21872611

[B75] Salomón C. Westermeier F. Puebla C. Arroyo P. Guzmán-Gutiérrez E. Pardo F. (2012). Gestational diabetes reduces adenosine transport in human placental microvascular endothelium, an effect reversed by insulin. *PLoS One* 7:e40578. 10.1371/journal.pone.0040578 22808198 PMC3395671

[B76] Schnermann J. (2015). Concurrent activation of multiple vasoactive signaling pathways in vasoconstriction caused by tubuloglomerular feedback: a quantitative assessment. *Annu. Rev. Physiol.* 77 301–322. 10.1146/annurev-physiol-021014-071829 25668021

[B77] Slugoski M. D. Smith K. M. Mulinta R. Ng A. M. Yao S. Y. Morrison E. L. (2008). A conformationally mobile cysteine residue (Cys-561) modulates Na+ and H+ activation of human CNT3. *J. Biol. Chem.* 283 24922–24934. 10.1074/jbc.M801793200 18621735 PMC3259814

[B78] Smith K. M. Ng A. M. Yao S. Y. Labedz K. A. Knaus E. E. Wiebe L. I. (2004). Electrophysiological characterization of a recombinant human Na+-coupled nucleoside transporter (hCNT1) produced in Xenopus oocytes. *J. Physiol.* 558(Pt 3), 807–823. 15194733 10.1113/jphysiol.2004.068189 PMC1665023

[B79] Sobrevia L. Abarzúa F. Nien J. K. Salomón C. Westermeier F. Puebla C. (2011). Review: differential placental macrovascular and microvascular endothelial dysfunction in gestational diabetes. *Placenta* 32(Suppl. 2), S159–S164. 10.1016/j.placenta.2010.12.011 21215450

[B80] Soler C. Felipe A. Mata J. F. Casado F. J. Celada A. Pastor-Anglada M. (1998). Regulation of nucleoside transport by lipopolysaccharide, phorbol esters, and tumor necrosis factor-alpha in human B-lymphocytes. *J. Biol. Chem.* 273 26939–26945. 10.1074/jbc.273.41.26939 9756942

[B81] Soler C. Valdés R. Garcia-Manteiga J. Xaus J. Comalada M. Casado F. J. (2001). Lipopolysaccharide-induced apoptosis of macrophages determines the up-regulation of concentrative nucleoside transporters Cnt1 and Cnt2 through tumor necrosis factor-alpha-dependent and -independent mechanisms. *J. Biol. Chem.* 276 30043–30049. 10.1074/jbc.M101807200 11346649

[B82] Ward J. L. Sherali A. Mo Z. P. Tse C. M. (2000). Kinetic and pharmacological properties of cloned human equilibrative nucleoside transporters, ENT1 and ENT2, stably expressed in nucleoside transporter-deficient PK15 cells. Ent2 exhibits a low affinity for guanosine and cytidine but a high affinity for inosine. *J. Biol. Chem.* 275 8375–8381. 10.1074/jbc.275.12.8375 10722669

[B83] Westermeier F. Salomón C. González M. Puebla C. Guzmán-Gutiérrez E. Cifuentes F. (2011). Insulin restores gestational diabetes mellitus-reduced adenosine transport involving differential expression of insulin receptor isoforms in human umbilical vein endothelium. *Diabetes* 60 1677–1687. 10.2337/db11-0155 21515851 PMC3114394

[B84] Yang C. Leung G. P. (2015). Equilibrative nucleoside transporters 1 and 4: which one is a better target for cardioprotection against ischemia-reperfusion injury? *J. Cardiovasc. Pharmacol.* 65 517–521. 10.1097/FJC.0000000000000194 26070128 PMC4461397

[B85] Yao S. Y. Ng A. M. Ritzel M. W. Gati W. P. Cass C. E. Young J. D. (1996). Transport of adenosine by recombinant purine- and pyrimidine-selective sodium/nucleoside cotransporters from rat jejunum expressed in Xenopus laevis oocytes. *Mol. Pharmacol.* 50 1529–1535. 8967974

[B86] Young J. D. (2016). The SLC28 (CNT) and SLC29 (ENT) nucleoside transporter families: a 30-year collaborative odyssey. *Biochem. Soc. Trans.* 44 869–876. 10.1042/BST20160038 27284054

[B87] Young J. D. Yao S. Y. Baldwin J. M. Cass C. E. Baldwin S. A. (2013). The human concentrative and equilibrative nucleoside transporter families, SLC28 and SLC29. *Mol. Aspects Med.* 34 529–547. 10.1016/j.mam.2012.05.007 23506887

[B88] Zamzow C. R. Xiong W. Parkinson F. E. (2008). Adenosine produced by neurons is metabolized to hypoxanthine by astrocytes. *J. Neurosci. Res.* 86 3447–3455. 10.1002/jnr.21789 18627033

[B89] Zhang D. Xiong W. Albensi B. C. Parkinson F. E. (2011). Expression of human equilibrative nucleoside transporter 1 in mouse neurons regulates adenosine levels in physiological and hypoxic-ischemic conditions. *J. Neurochem.* 118 4–11. 10.1111/j.1471-4159.2011.07242.x 21395582

[B90] Zimmerman M. A. Tak E. Ehrentraut S. F. Kaplan M. Giebler A. Weng T. (2013). Equilibrative nucleoside transporter (ENT)-1-dependent elevation of extracellular adenosine protects the liver during ischemia and reperfusion. *Hepatology* 58 1766–1778. 10.1002/hep.26505 23703920 PMC3795856

